# Supplementary material for: Tunable assembly of truncated nanocubes by evaporation-driven poor-solvent enrichment
Source: Nat Commun. 2019 Sep 17;10:4228. doi: 10.1038/s41467-019-12237-y (PMC6748999; doi:10.1038/s41467-019-12237-y)
Supplement: Supplementary file 1 — Supplementary Information [file 41467_2019_12237_MOESM1_ESM.pdf]

Supplementary Information for

Tunable assembly of truncated nanocubes by evaporation-driven poor-solvent enrichment

Lv et al.

Contents

Supplementary Figures 2

Supplementary Figure 1. 2
Supplementary Figure 2. 3
Supplementary Figure 3. 3
Supplementary Figure 4. 4
Supplementary Figure 5. 4
Supplementary Figure 6. 5
Supplementary Figure 7. 5
Supplementary Figure 8. 6
Supplementary Figure 9. 7
Supplementary Figure 10. 7
Supplementary Figure 11. 8
Supplementary Figure 12. 8
Supplementary Figure 13. 9
Supplementary Figure 14. 9
Supplementary Figure 15. 10
Supplementary Figure 16. 10
Supplementary Figure 17. 11
Supplementary Figure 18. 11

Supplementary Tables 12

Supplementary Table 1 12
Supplementary Table 2 13

Supplementary Notes 14

Supplementary Note 1: Composition of nanocubes before and after purification 14
Supplementary Note 2: Evaporation model of poor-solvent enrichment 14
Supplementary Note 3: Correlation of $H(0)$, $H(t_1)$ and $H(t_2)$ to Δt, c_C, and P_C 17
Supplementary Note 4: Up and down scaling and minimization of assembly time 18
Supplementary Note 5: Peak assignment of SAXS profile and structure analysis 19

Supplementary References 20

Supplementary Figures

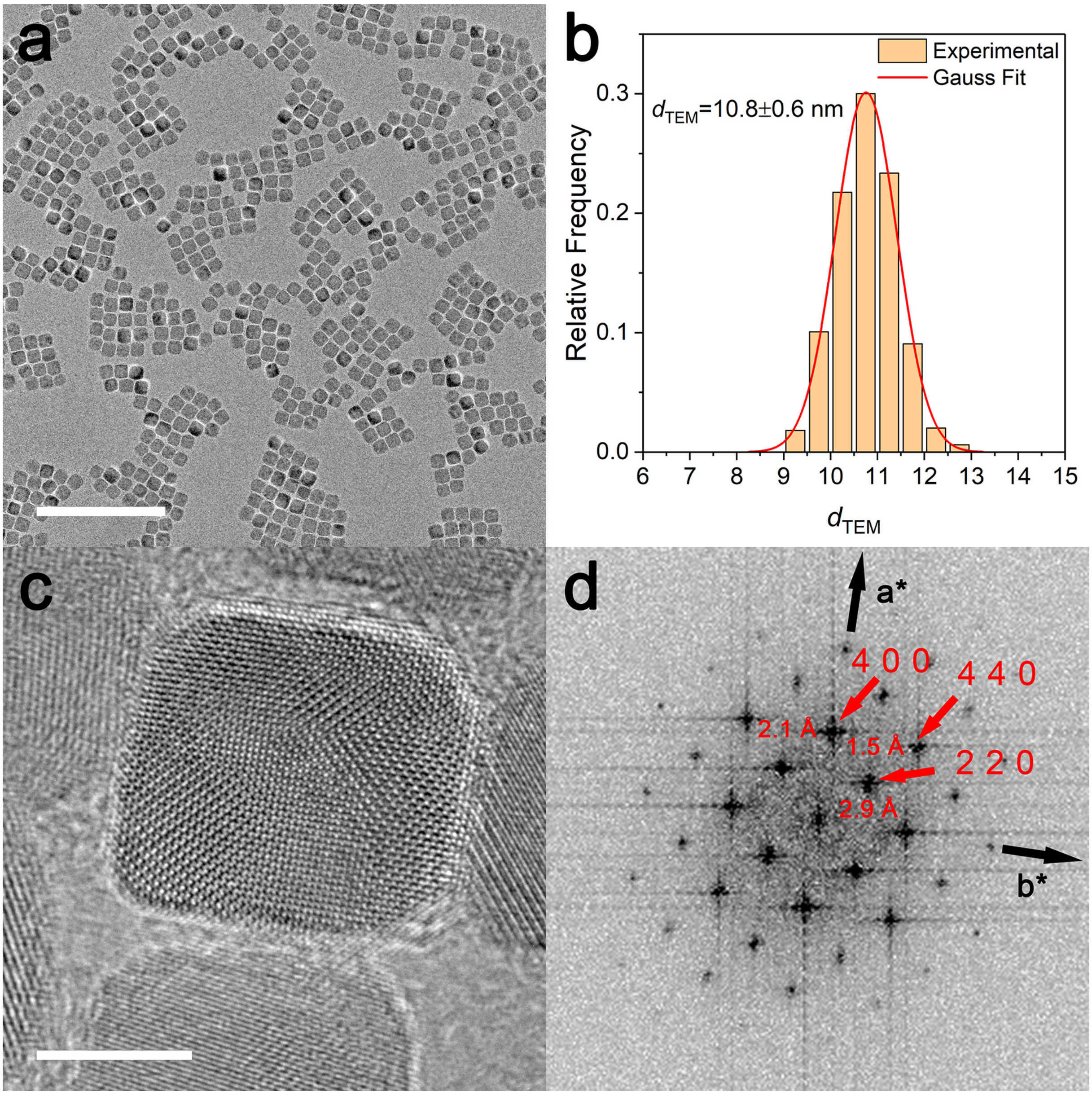

Supplementary Figure 1. Structure information of truncated iron oxide nanocubes. a TEM image of the purified truncated NCs. **b** Histogram of the particle edge length d_{TEM} and the corresponding Gaussian fit. The edge length has been measured of 500 individual NCs with the program Nano Measurer. **c** HRTEM image of a truncated NC and **d** corresponding FFT pattern. The *d*-spacing obtained from the FFT pattern fits the *d*-spacing of magnetite very well where 2.96 Å for (220), 2.09 Å for (400), and 1.48 Å for (440) were obtained.

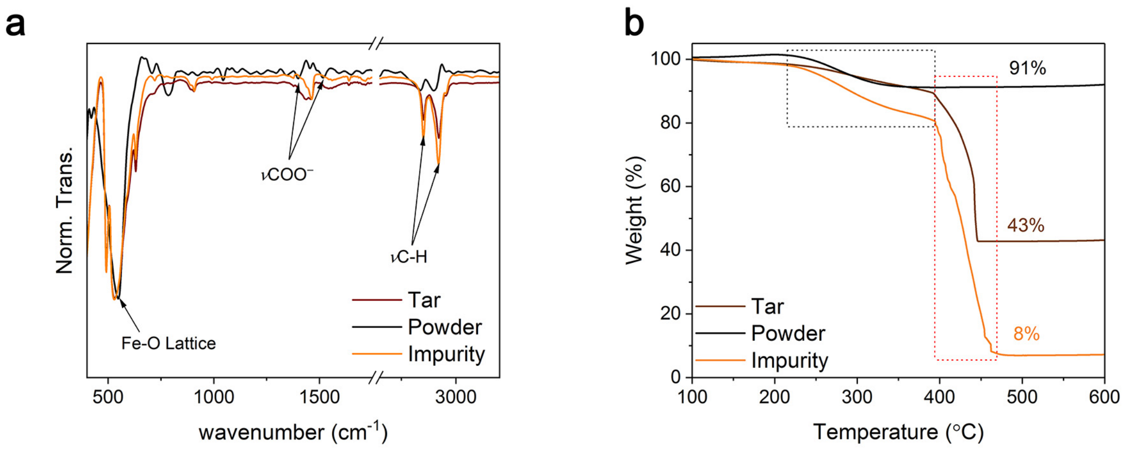

Supplementary Figure 2. Composition of nanocubes before and after purification. **a** FTIR and **b** TG data of original tar (brown curves), purified NC powder (black curves), and impurities (orange curves).

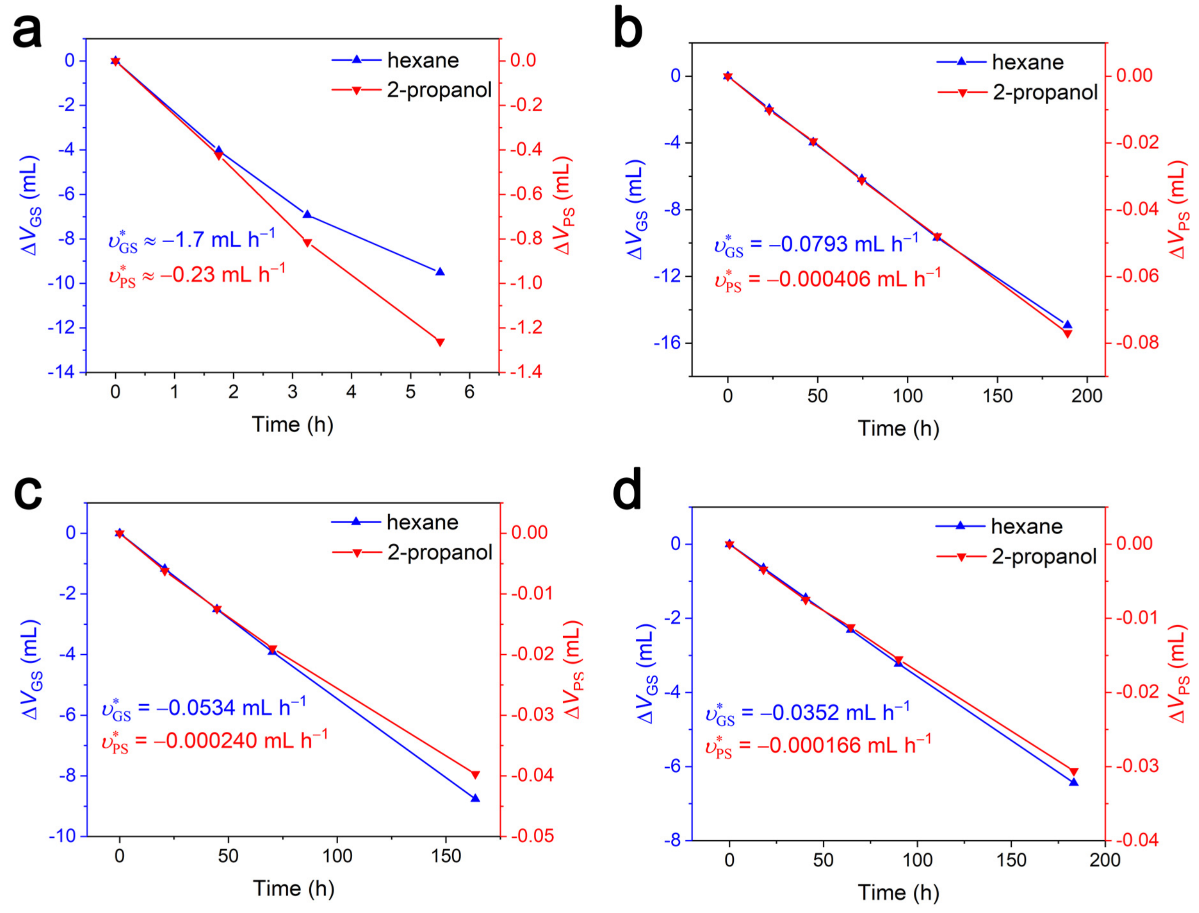

Supplementary Figure 3. Solvent evaporation rate measurements of vessels with different number of PE layers. Evaporation rates of pure hexane and 2-propanol for vessels with **a** $N_{PE} = 0$; **b** $N_{PE} = 1$; **c** $N_{PE} = 2$ and; **d** $N_{PE} = 4$.

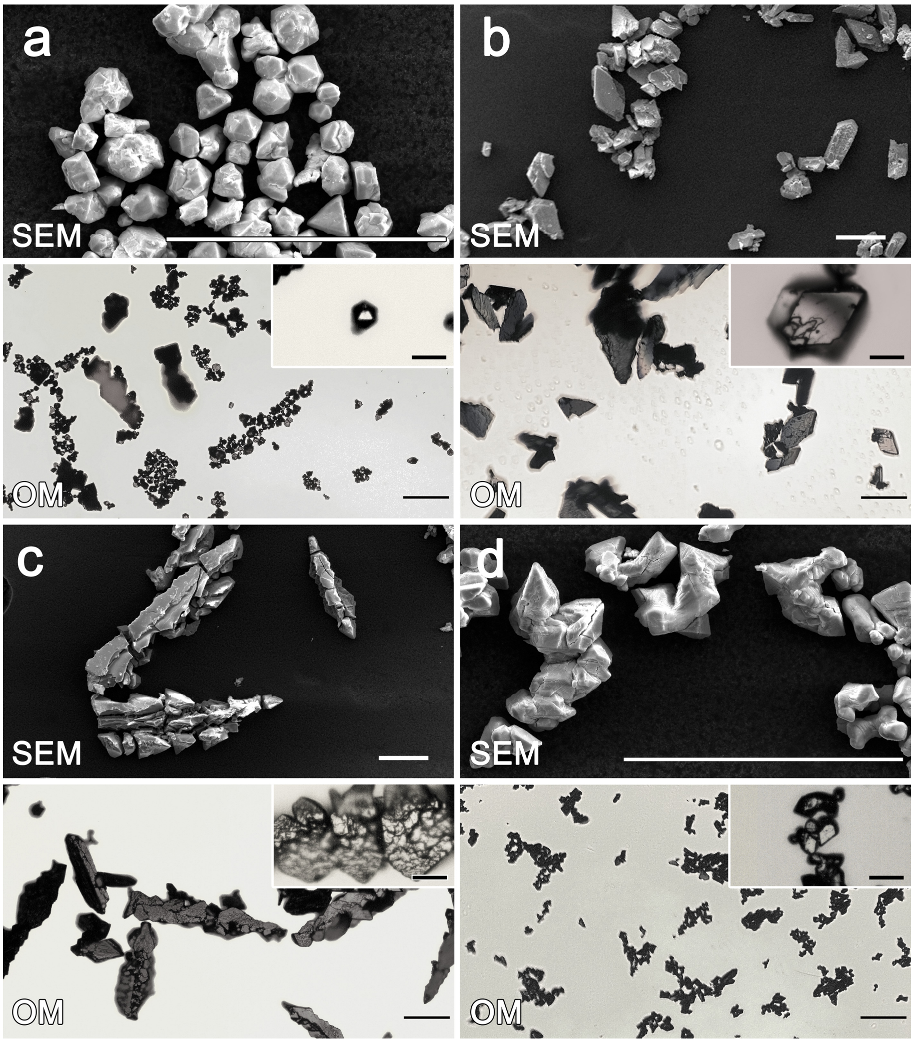

Supplementary Figure 4. Morphological study of mesocrystals with different V_{GS}/V_{PS} values. SEM and OM images of multiple mesocrystals for samples; **a** #1 (V_{GS}/V_{PS} = 2.5); **b** #2 (V_{GS}/V_{PS} = 5); **c** #3 (V_{GS}/V_{PS} = 12.5); **d** #4 ($V_{GS}/V_{PS} = \infty$). Scale bar = 100 μm for SEM, = 100 μm for OM, and = 20 μm for OM inset.

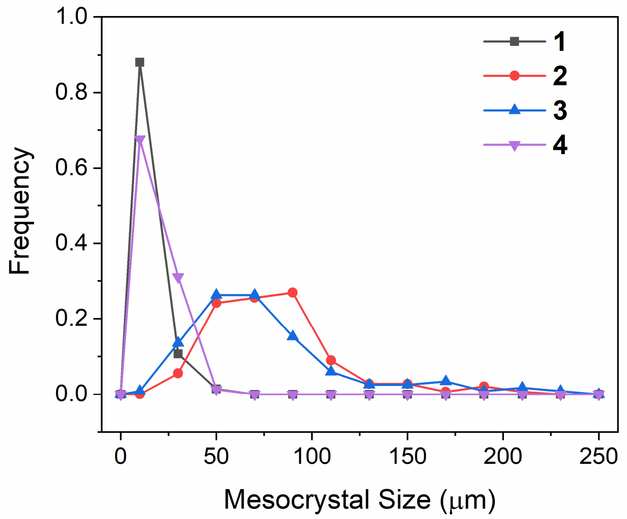

Supplementary Figure 5. Size distributions based on SEM image analysis of mesocrystals with different V_{GS}/V_{PS} values. Sample #**1** (V_{GS}/V_{PS} = 2.5); sample #**2** (V_{GS}/V_{PS} = 5); sample #**3** (V_{GS}/V_{PS} = 12.5); sample #**4** ($V_{GS}/V_{PS} = \infty$). Bin size = 20 μm.

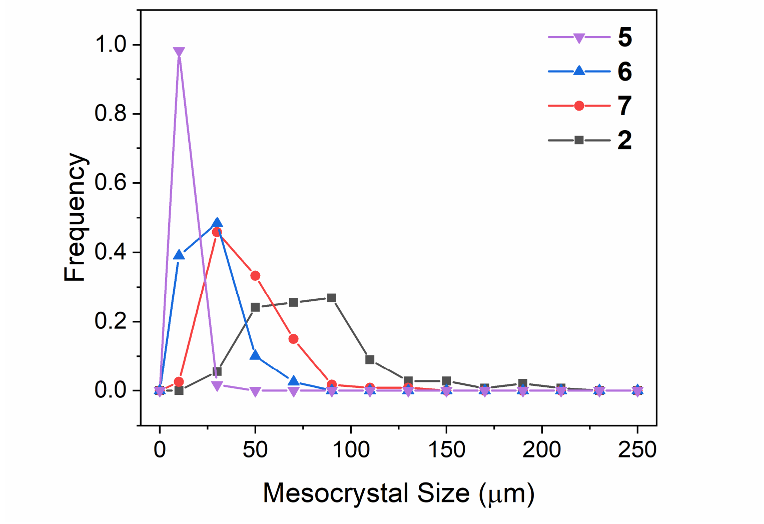

Supplementary Figure 6. Size distributions based on SEM image analysis of mesocrystals with different *c*(0) values. Sample #**5** (*c*(0) = 0.375); sample #**6** (*c*(0) = 0.75); sample #**7** (*c*(0) = 1.5); sample #**2** (*c*(0) = 3.0). Bin size = 20 μm.

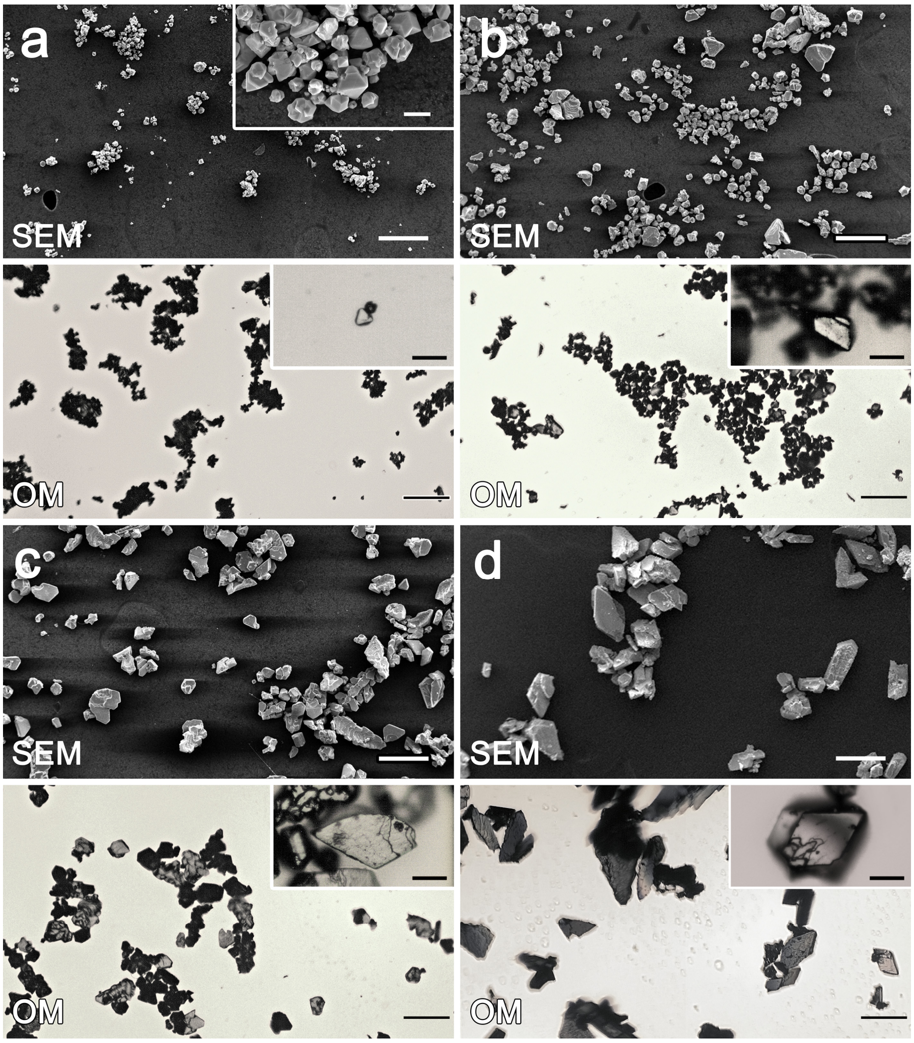

Supplementary Figure 7. Morphological study of mesocrystals with different *c*(0) values. SEM and OM images of multiple mesocrystals for samples; **a** #**5** (*c*(0) = 0.375); **b** #**6** (*c*(0) = 0.75); **c** #**7** (*c*(0) = 1.5) and **d** #**2** (*c*(0) = 3.0). Scale bar = 100 μm for SEM, = 10 μm for SEM inset, = 100 μm for OM, and = 20 μm for OM inset.

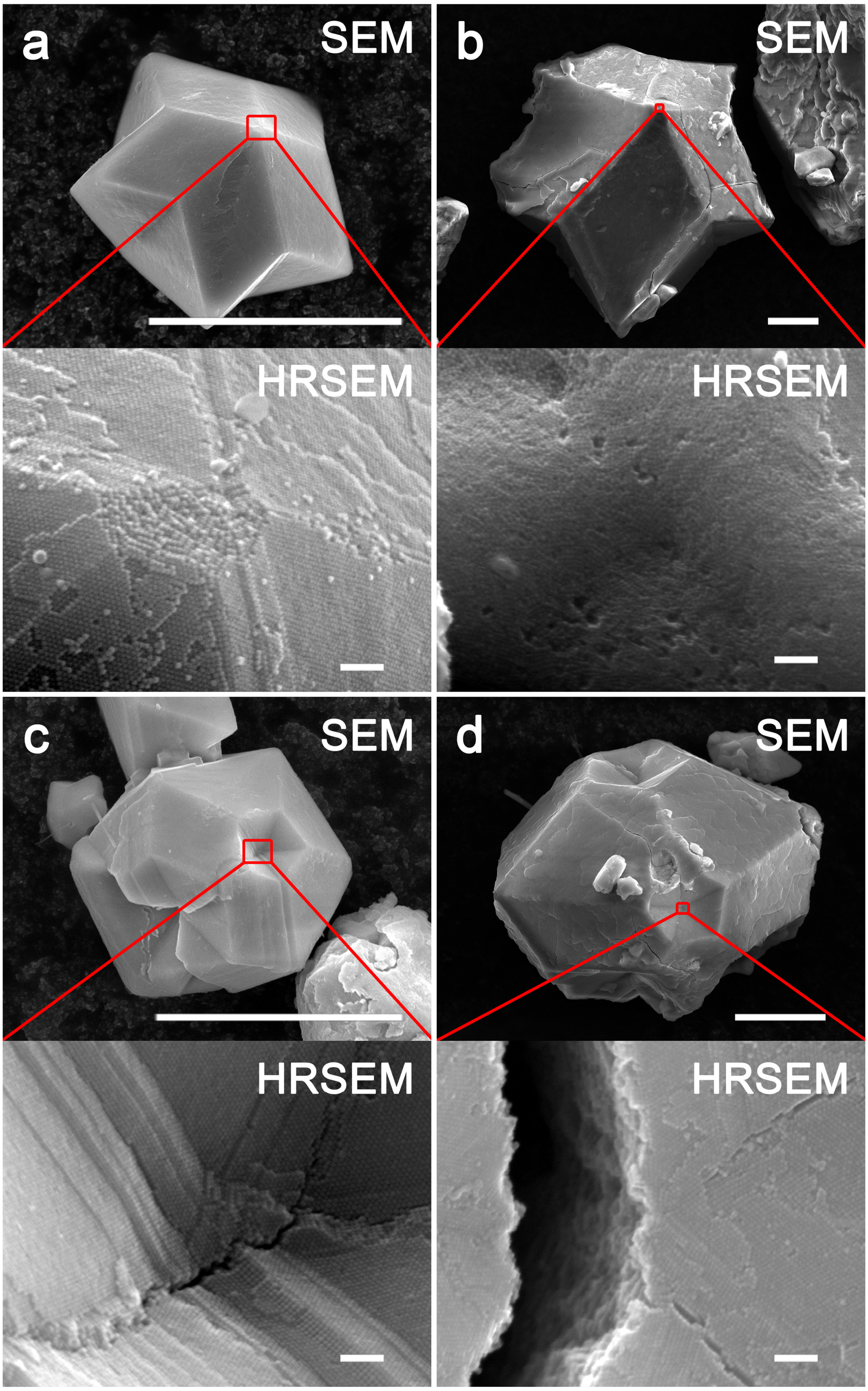

Supplementary Figure 8. Morphological study of mesocrystals with five-armed star and quasi-icosahedral morphologies. SEM and HRSEM images of mesocrystals with five-armed star morphology in samples; **a** #**5** and; **b** #**2**. The HRSEM image of the mesocrystal vertex of sample #**5**, depict the boundaries of five different domains. The HRSEM image of the mesocrystal vertex of sample #**2** shows that the NC alignment was almost random. SEM and HRSEM images of mesocrystals with quasi-icosahedral morphology of; **c** sampe #**5** and; **d** sample #**2**. The HRSEM image of the mesocrystal vertex in sample #**5** shows the five domains and a crack. The domain boundaries of the mesocrystal vertex of sample #**2** are less clear. Scale bar = 10 μm for SEM, and = 100 nm for HRSEM.

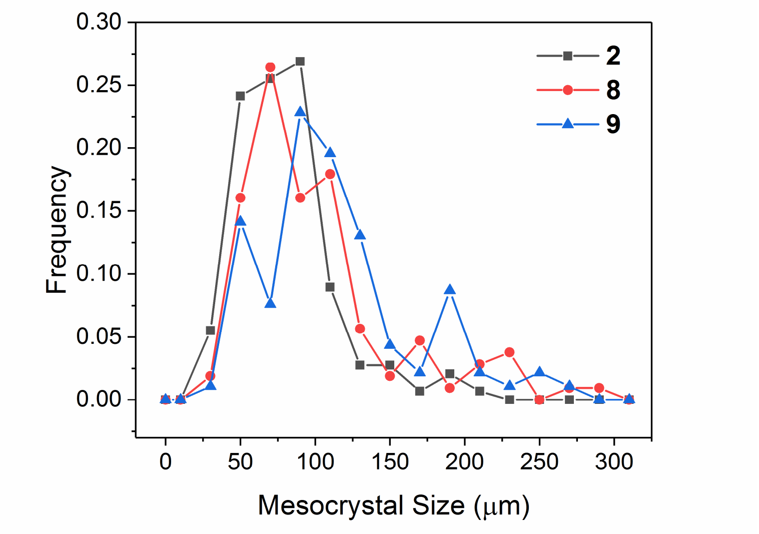

Supplementary Figure 9. Size distributions from SEM images of mesocrystals with different N_{PE} values. Sample #**2** (N_{PE} = 1); sample #**8** (N_{PE} = 2); sample #**9** (N_{PE} = 4). Bin size 20 = μm.

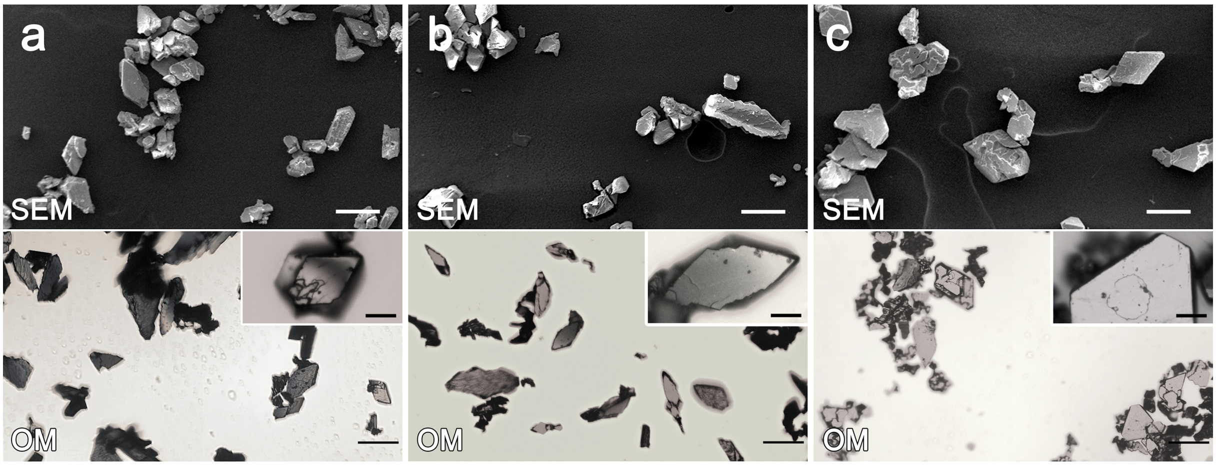

Supplementary Figure 10. Morphological study of mesocrystals with different N_{PE} values. SEM (top) and OM (bottom) images of multiple mesocrystals for samples; **a** #**2** (N_{PE} = 1); **b** #**8** (N_{PE} = 2), and; **c** #**9** (N_{PE} = 4). Scale bar = 100 μm for SEM, = 100 μm for OM, and = 20 μm for OM inset.

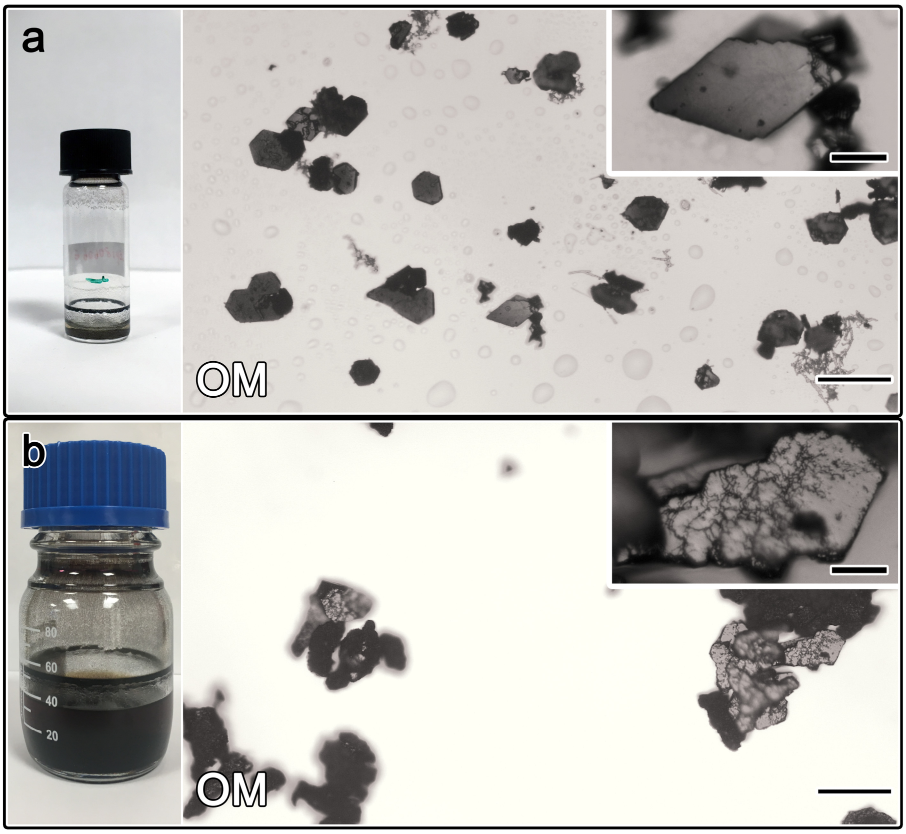

Supplementary Figure 11. Morphological study of mesocrystals with different initial volumes. The vessels and OM images of **a** sample #**10** ($V(0)$ = 1.5 mL) and **b** sample #**11** ($V(0)$ = 120 mL) with different initial volumes. $H(0)$, $H(t_1)$ and $H(t_2)$ values can be measured from the green line and the black ring on the vessel wall or read directly from the scales on the vessel wall. Scale bar = 100 μm for OM, and = 20 μm for OM inset.

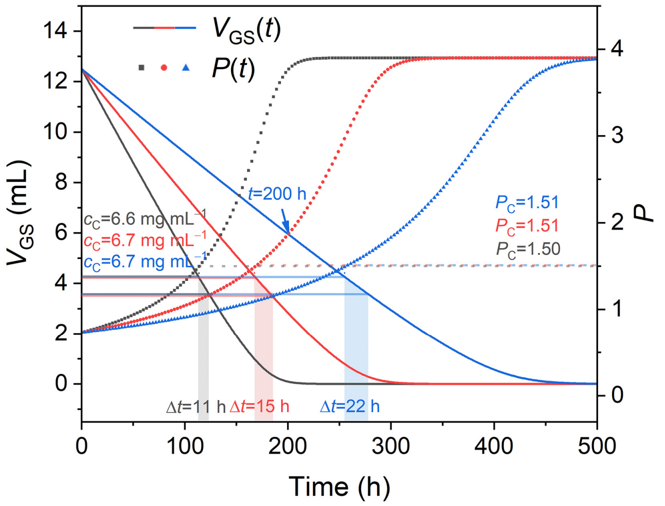

Supplementary Figure 12. Time-dependent evaporation and polarity modeling of solvent mixtures with different N_{PE} values. The $V_{GS}(t)$ (solid lines linked to left and bottom axis) and $P(t)$ (dot lines linked to right and bottom axis) curves from modeling for $V_{GS}/V_{PS} = 5$, $N_{PE} = 1$ (black), $V_{GS}/V_{PS} = 5$, $N_{PE} = 2$ (red), and $V_{GS}/V_{PS} = 5$, $N_{PE} = 4$ (blue). The auxiliary lines and shades show the relationship between experimental $V_{GS}(t_1)$, $V_{GS}(t_2)$, t_1, t_2, and P_C for mesocrystals produced from dispersions of different N_{PE}; sample #**2** (black), sample #**8** (red), and sample #**9**/#**12** (blue). Corresponding Δt, $c_{C,}$ and P_C values were also given.

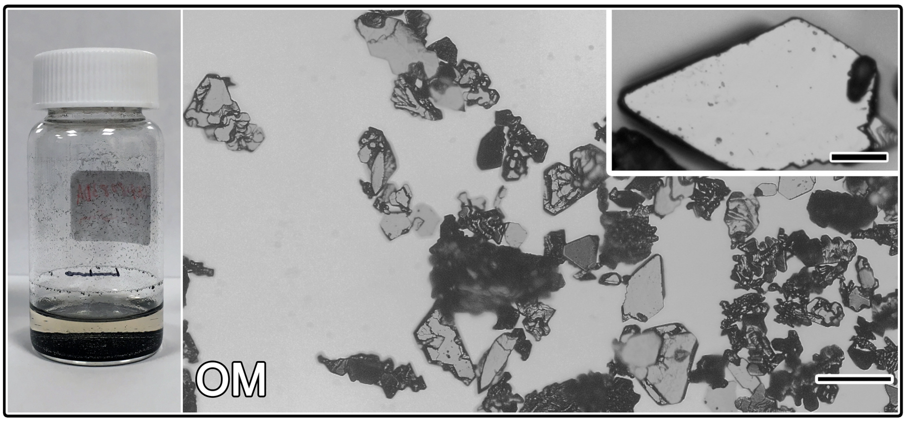

Supplementary Figure 13. Morphological study of mesocrystals with smaller initial volume. The vessel and OM images of EDPSE assembly of sample #**12**. $H(0)$, $H(t_1)$ and $H(t_2)$ values can be measured from the green line and the black ring on the vessel wall. Scale bar = 100 μm for OM, and = 20 μm for OM inset.

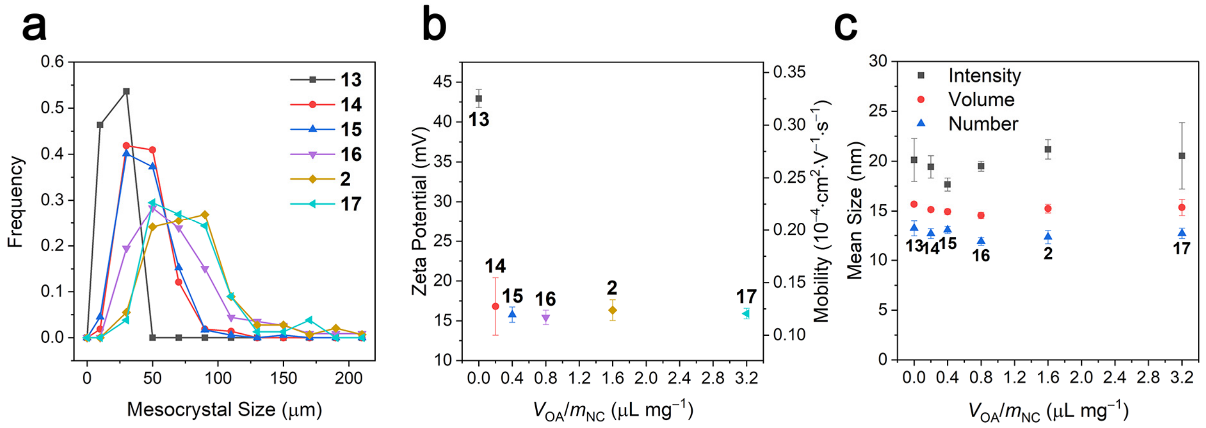

Supplementary Figure 14. Size and surface charge study of mesocrystals/nanocubes with different V_{OA}/m_{NC} values. a Size distribution of mesocrystals obtained from SEM images of samples #**13** (V_{OA}/m_{NC} = 0 μL mg^{-1}), #**14** (V_{OA}/m_{NC} = 0.2 μL mg^{-1}); #**15** (V_{OA}/m_{NC} = 0.4 μL mg^{-1}); #**16** (V_{OA}/m_{NC} = 0.8 μL mg^{-1}), #**2** (V_{OA}/m_{NC} = 1.6 μL mg^{-1}), and; #**17** (V_{OA}/m_{NC} = 3.2 μL mg^{-1}). Bin size 20 μm. **b** Zeta potential (left axis) and corresponding electrophoretic mobility (right axis) of oleate-capped nanocubes for samples #**13–16**, #**2**, and #**17** with different V_{OA}/m_{NC} ratios. Error bars represent the standard deviation of the mean of three measurements. **c** Hydrodynamic size of dispersions of oleate-capped nanocubes of samples #**13–16**, #**2** and #**17** with different V_{OA}/m_{NC} values. Error bars represent the standard deviation of the mean of three measurements.

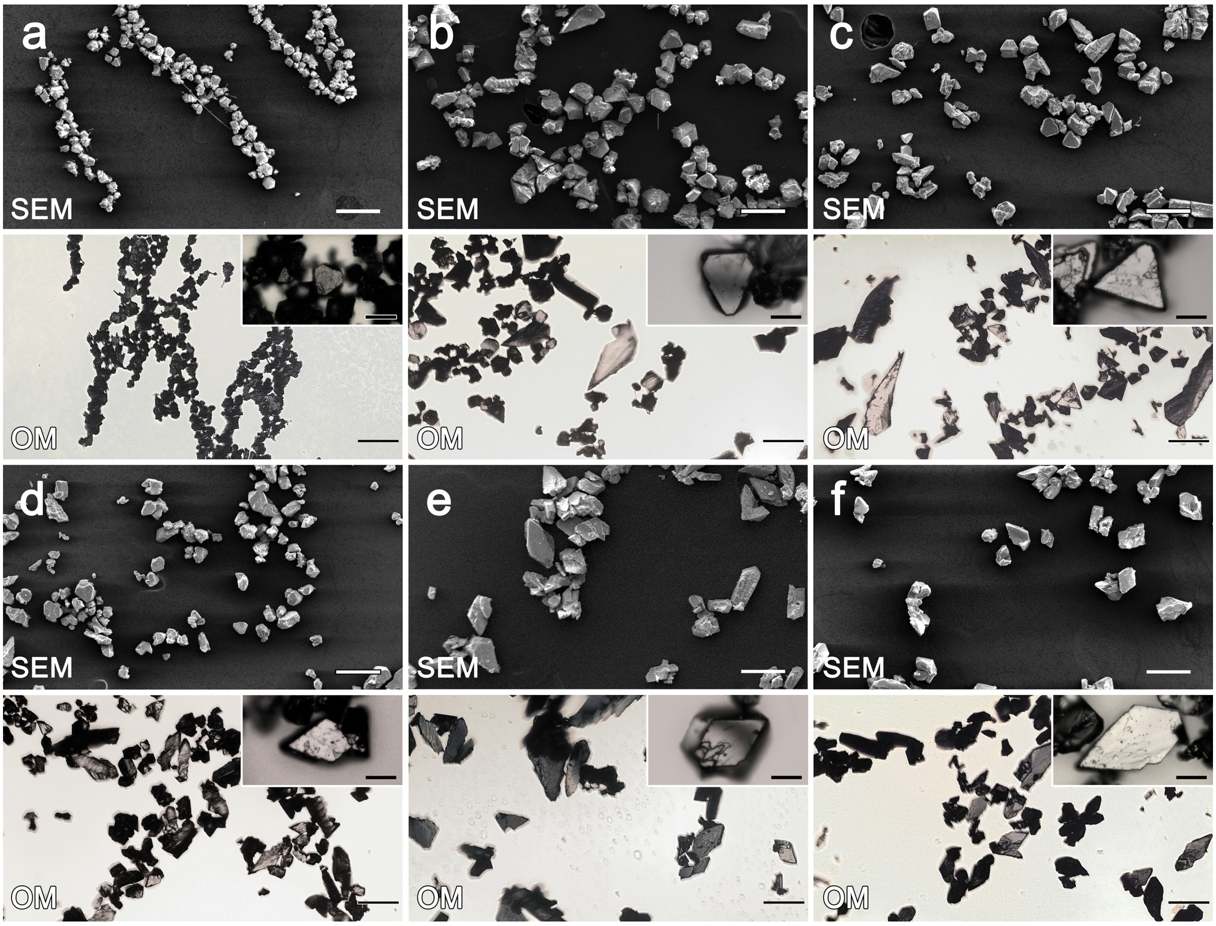

Supplementary Figure 15. Morphological study of mesocrystals with different V_{OA}/m_{NC} values. SEM and OM images of multiple mesocrystals for samples; **a** #**13** (V_{OA}/m_{NC} = 0 µL mg^{-1}), **b** #**14** (V_{OA}/m_{NC} = 0.2 µL mg^{-1}); **c** #**15** (V_{OA}/m_{NC} = 0.4 µL mg^{-1}); **d** #**16** (V_{OA}/m_{NC} = 0.8 µL mg^{-1}), **e** #**2** (V_{OA}/m_{NC} = 1.6 µL mg^{-1}), and; **f** #**17** (V_{OA}/m_{NC} = 3.2 µL mg^{-1}). Scale bar = 100 µm for SEM, = 100 µm for OM, and = 20 µm for OM inset.

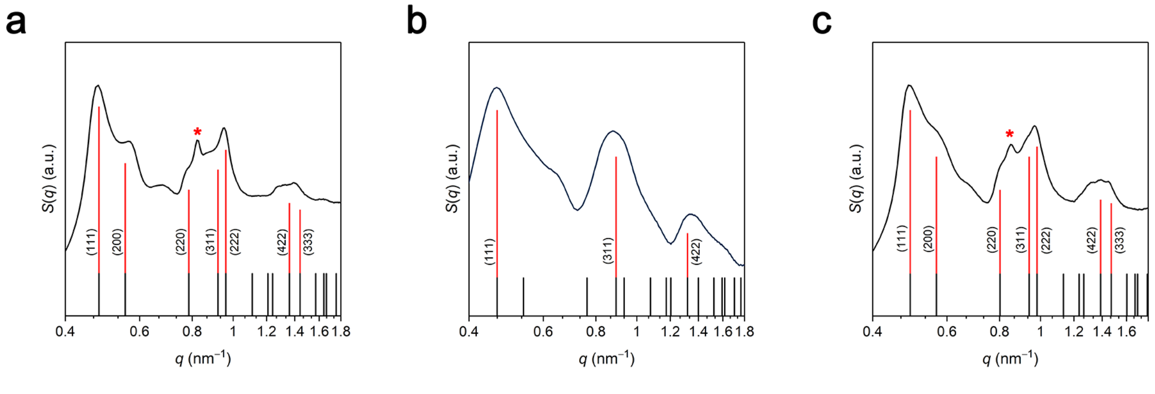

Supplementary Figure 16. SAXS curves of mesocrystals obtained from drying droplets with different V_{GS}/V_{PS} and V_{OA}/m_{NC} values. The structure factor $S(q)$ (black curve), obtained from a 1D SAXS curve, of samples **a** #**D1** (V_{GS}/V_{PS} = 5, V_{OA}/m_{NC} = 1.6 µL mg^{-1}), **b** #**D2** (V_{GS}/V_{PS} = ∞, V_{OA}/m_{NC} = 1.6 µL mg^{-1}), and **c** #**D3** (V_{GS}/V_{PS} = 5, V_{OA}/m_{NC} = 0 µL mg^{-1}) with indexed reflections of an *fcc* superlattice (red long vertical lines and black short vertical lines indicate the visible and allowed indices of an *fcc* superlattice, respectively). The frames used for indexing were recorded after major crystal growth ceased at 214 s for #**D1**, 363 s for #**D2**, and 244 s for #**D3**. Peaks marked with a red asterisk in (**a**) and (**c**) cannot be assigned to an *fcc* lattice. We assume it originates from a distorted phase.[8]

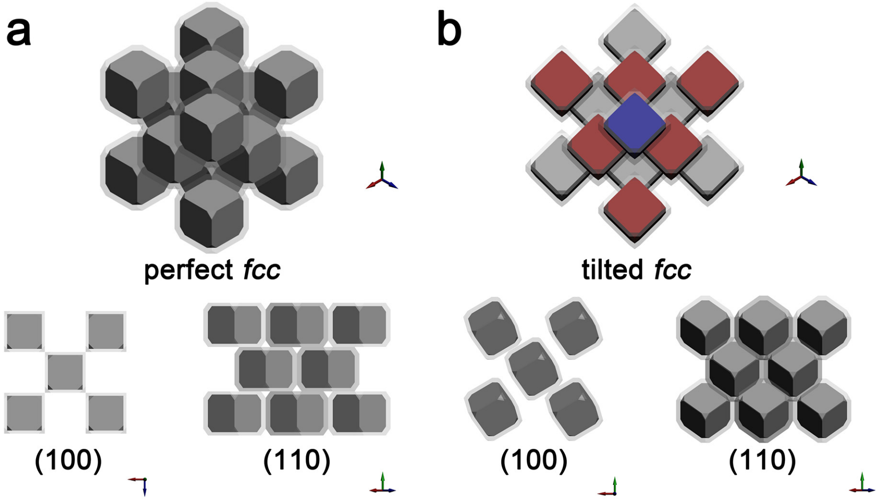

Supplementary Figure 17. Schematic illustration of *fcc* unit cells with different packing manners. **a** perfect *fcc* unit cell and **b** tilted *fcc* unit cell (right) of truncated NCs viewed along the [111] superlattice direction (top). The (100) and (110) superlattice facets of the two lattices are also displayed (bottom). The *x*, *y* and *z* axes were indicated as red, blue and green arrows, respectively.

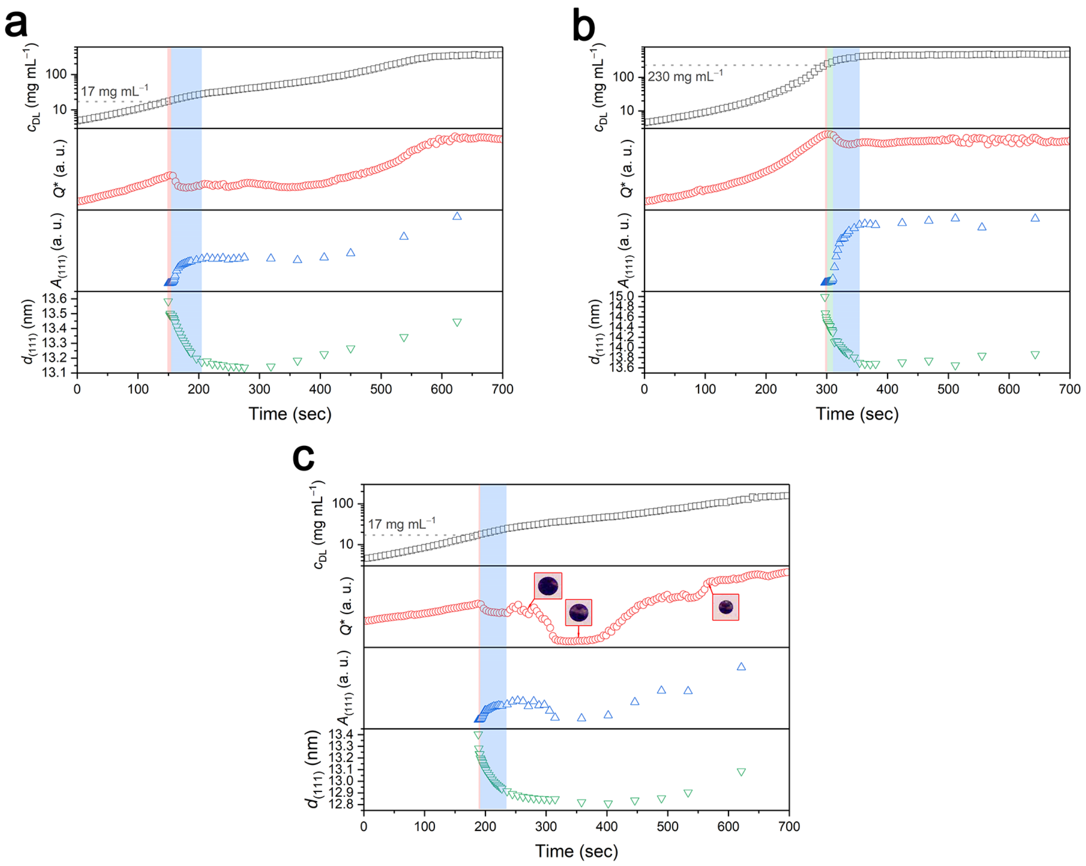

Supplementary Figure 18. Time-resolved SAXS measurements of assembly of mesocrystals by evaporation-driven poor-solvent enrichment in a levitating droplet. Full data of $c_{DL}(t)$ (black), $Q^*(t)$ (red), $A_{(111)}(t)$ (blue) and $d_{(111)}(t)$ (green) curves for the whole measurement for samples **a** #**D1** ($V_{GS}/V_{PS} = 5$, $V_{OA}/m_{NC} = 1.6\ \mu L\ mg^{-1}$), **b** #**D2** ($V_{GS}/V_{PS} = \infty$, $V_{OA}/m_{NC} = 1.6\ \mu L\ mg^{-1}$), and **c** #**D3** ($V_{GS}/V_{PS} = 5$, $V_{OA}/m_{NC} = 0\ \mu L\ mg^{-1}$). The shaded regions indicate nucleation (red), extended nucleation and early crystal growth (green), and major crystal growth (blue). The images of the droplet in (**c**) show a phase separation after ~250 s, which lead to a decreasing signal intensity of Q^* and $A_{(111)}$. After ~400s, the signal intensity increased again as the formed mesocrystals were irradiated by the X-ray beam.

Supplementary Tables

Supplementary Table 1. Analytical expression of $V_{GS}(t)$ for different V_{GS}/V_{PS} and N_{PE} values.

V_{GS}/V_{PS}	N_{PE}	$V_{GS}(0)$ (mL)	$V_{PS}(0)$ (mL)	υ_{GS}^{*} (mL h^{-1})	$\frac{dV_{GS}}{dt}$ (mL h^{-1})	$V_{GS}(t)$ (mL)
2.5	1	10.7	4.3	−0.0793	$-0.0793\frac{V_{GS}}{V_{GS}+1.22}$	$1.22\times \text{lambertW}(5.95\times 10^{4}\times e^{-0.0650t})$
5	1	12.5	2.5	−0.0793	$-0.0793\frac{V_{GS}}{V_{GS}+0.708}$	$0.708\times \text{lambertW}(8.21\times 10^{8}\times e^{-0.112t})$
12.5	1	13.9	1.1	−0.0793	$-0.0793\frac{V_{GS}}{V_{GS}+0.311}$	$0.311\times \text{lambertW}(1.15\times 10^{21}\times e^{-0.255t})$
∞	1	15	0	−0.0793	−0.0793	$15-0.0793t$
5	2	12.5	2.5	−0.0534	$-0.0534\frac{V_{GS}}{V_{GS}+0.708}$	$0.708\times \text{lambertW}(8.21\times 10^{8}\times e^{-0.0754t})$
5	4	12.5	2.5	−0.0352	$-0.0352\frac{V_{GS}}{V_{GS}+0.708}$	$0.708\times \text{lambertW}(8.21\times 10^{8}\times e^{-0.0497t})$
4	1	12	3	−0.0793	$-0.0793\frac{V_{GS}}{V_{GS}+0.849}$	$0.849\times \text{lambertW}(1.94\times 10^{7}\times e^{-0.0934t})$

a The lambertW is the Lambert W function.

Supplementary Table 2. Important quantities obtained for samples #**1−17** in dependence of their growth parameters.

Sample #	V_{GS}/V_{PS}	$c(0)$ (mg mL^{-1})	N_{PE}	V_{OA}/m_{NC} (μL mg^{-1})	$H(0)$ (mm)	$H(t_1)$ (mm)	$H(t_2)$ (mm)	$V_{GS}(t_1)$ (mL)	$V_{GS}(t_2)$ (mL)	t_1 (h)	t_2 (h)	Δt (h)	c_C (mg mL^{-1})	P_C	x_{OA}[c] (%)	m_{NC}/A_B (mg cm^{-2})
1	2.5	3.0	1	1.6	30.6	23.3	20.7	7.13	5.85	52	71	19	3.9	1.53	0.6	9.2
2	5	3.0	1	1.6	31.1	14.1	12.6	4.30	3.58	113	124	11	6.6	1.50	1.1	9.3
3	12.5	3.0	1	1.6	30.4	7.0	6.3	2.34	2.00	153	158	5	13	1.31	2.1	9.1
4[a]	∞	3.0	1	1.6	30.7	1.0	--	0.49	--	183	--	--	90	0.1	15.1	9.2
5	5	0.375	1	1.6	31.8	12.3	11.7	3.30	3.02	128	132	4	1.0	1.74	0.2	1.2
6	5	0.75	1	1.6	32.9	13.5	12.6	3.66	3.24	123	129	6	1.8	1.64	0.3	2.5
7	5	1.5	1	1.6	32.1	13.7	12.6	3.90	3.39	119	127	8	3.5	1.58	0.6	4.8
8	5	3.0	2	1.6	27.9	12.5	11.2	4.22	3.52	170	185	15	6.7	1.51	1.1	8.4
9	5	3.0	4	1.6	31.3	14.0	12.7	4.26	3.59	256	278	22	6.7	1.51	1.1	9.4
10	5	3.0	4	1.6	13.1	5.9	5.1	0.43	0.33	--	--	--	6.7	1.47	1.1	3.9
11[b]	5	3.0	1	1.6	--	--	--	37	31	--	--	--	6.3	1.43	1.0	14.9
12	2.4	5.3	4	1.6	16.5	13.1	11.8	4.25	3.58	56	79	23	6.7	1.51	1.1	8.7
13	5	3.0	1	0	31.1	14.1	12.7	4.30	3.63	113	123	10	6.6	1.50	0	9.3
14	5	3.0	1	0.2	29.6	13.1	11.8	4.14	3.48	115	125	10	6.8	1.53	0.1	8.9
15	5	3.0	1	0.4	31.1	14.3	12.6	4.40	3.58	111	123	12	6.5	1.48	0.3	9.3
16	5	3.0	1	0.8	30.1	13.5	12.2	4.23	3.58	114	124	10	6.7	1.51	0.5	9.0
17	5	3.0	1	3.2	30.6	14.1	12.4	4.41	3.58	111	123	12	6.5	1.47	2.1	9.2
X	4	8	1	1.6	31.0	18.7	15.4	6.05	4.45	82	106	24	13.3	1.36	2.1	24.8

a The measuring error for $H(t_1)$ was larger, since the crystallization ring on the vessel wall was not very clear. P for this sample is always 0.1 regardless of the influence of OA on the polarity.

b The V_{GS} values were read directly from the vessel scale as shown in Supplementary Figure 11b.

c The free OA volume fraction x_{OA} at the onset of mesocrystal formation is obtained simply by dividing V_{OA} with $V(t_1)$. It should be noticed that a small amount of OA (ca. 0.05−0.08 μL OA per mg NCs) will bond to NC surface when adding OA to the purified NC, which will result in a reduction of the x_{OA} value. However, for most of our samples where V_{OA}/m_{NC} = 1.6 μL mg^{-1}, the is estimated to be less than 5%.

Supplementary Notes

Supplementary Note 1: Composition of nanocubes before and after purification

The Fourier transform infrared (FTIR) spectra in Supplementary Figure 2a were normalized to the intensity of the $\nu_{\text{Fe-O}}$ lattice band around 500 cm^{-1}. Characteristic $\nu_{\text{C-H}}$ bands of hydrocarbon chains at 2838−2920 cm^{-1} can be observed in the impurities, tar, and NC powder with a decreasing intensity. In the purified NC powder, the peak at 1710 cm^{-1} of unbound carboxylic groups was absent, and the asymmetric and symmetric vibration modes of COO^- at 1397 cm^{-1} and 1517 cm^{-1} respectively were observed, indicating only chemically bonded OA on the iron oxide surface.[1] In the thermogravimetry (TG) curves in Supplementary Figure 2b, the tar-like product, only treated with a toluene/ethanol washing process, contains 43 wt% of iron oxide, indicating that this tar contains besides surface bound OA on the NCs, free OA, or solvents (ca. 10 wt%, decomposed from 210 to 390 °C, black dash-line box), also high decomposition temperature impurities (ca. 47 wt%, decomposed from 390 to 450 °C, red dash-line box). The high decomposition temperature of the impurities indicates a formation of larger hydrocarbon compounds during the thermal decomposition reaction. After washing with 1-pentanol, the impurities were fully removed, leaving a black powder containing 91 wt% of iron oxide and 9 wt% of coordinated OA on the NC surfaces, corresponding to an OA coverage of 2.0 molecules nm^{-2}. It should be noticed that after removal of the impurities, the NCs became more sensitive to oxidation, since their weight increased visibly with increasing temperature. The impurities contain only 8 wt% iron oxide, indicating the efficiency of the 1-pentanol washing step, which kept a very high amount of NCs in the purified powder. Both FTIR and TG indicate an efficient removal of excessive ligand and impurities from the original tar.

Supplementary Note 2: Evaporation model of poor-solvent enrichment

In an isothermal quasi equilibrium system, the diffusion rate of solvent 1, $\mathrm{d}V_1/\mathrm{d}t$ through the membrane is directly proportional to its partial pressure difference on the two sides of the membrane, which satisfies Fick’s first law,[2]

$$\frac{\mathrm{d}V_1}{\mathrm{d}t} = PA_0(p_1 - p_1^0) \qquad (1)$$

where V_1 is the volume of solvent 1, t is the time, p_1 and p_1^0 are the partial pressures of component 1 inside and outside the vessel, respectively, which were separated by the membrane.

P and A_O are the permeability constant and vessel opening area, respectively. With $p_1^0 \approx 0$, the diffusion rate of component 1 can be expressed as:

$$v_1 = \frac{\mathrm{d}V_1}{\mathrm{d}t} = PA_Op_1 \qquad (2)$$

Based on Supplementary Equation 2, the evaporation rate of pure hexane v_{GS}^* at the saturated vapor pressure p_{GS}^* can be expressed as:

$$v_{GS}^* = PA_Op_{GS}^* \qquad (3)$$

Combining Supplementary Equation 2 and 3 yields the evaporation rate of hexane at arbitrary vapor pressure p_{GS}:

$$\frac{\mathrm{d}V_{GS}}{\mathrm{d}t} = v_{GS}^* \frac{p_{GS}}{p_{GS}^*} \qquad (4)$$

In the hexane–2-propanol binary system, the phase equilibrium diagram at isothermal condition (298 K) can be obtained from UNIQUAC (universal quasichemical) activity coefficient model calculation, as the phase equilibrium diagram at isothermal condition (298 K) in the hexane–2-propanol binary system illustrates in the diagram below.[3-5]

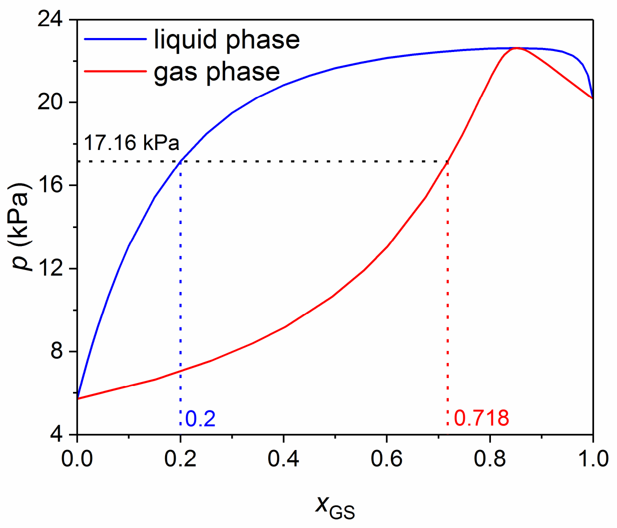

The blue and red curve correspond to the mole fraction of hexane in liquid and gas phase at a certain total vapor pressure, respectively. For example, as the dotted lines indicate, when x_{GS} is 0.15 in the solvent mixture, the total vapor pressure is 15.42 kPa and x_{GS} becomes 0.675 in the gas phase. Therefore the partial vapor pressure of hexane is $p_{GS} = 15.42 \times 0.675 = 10.41$ kPa. We can convert it into the relationship between p_{GS}/p_{GS}^* and the volume of hexane and 2-propanol (V_{GS} and V_{PS}), as the black curve shows in the following diagram.

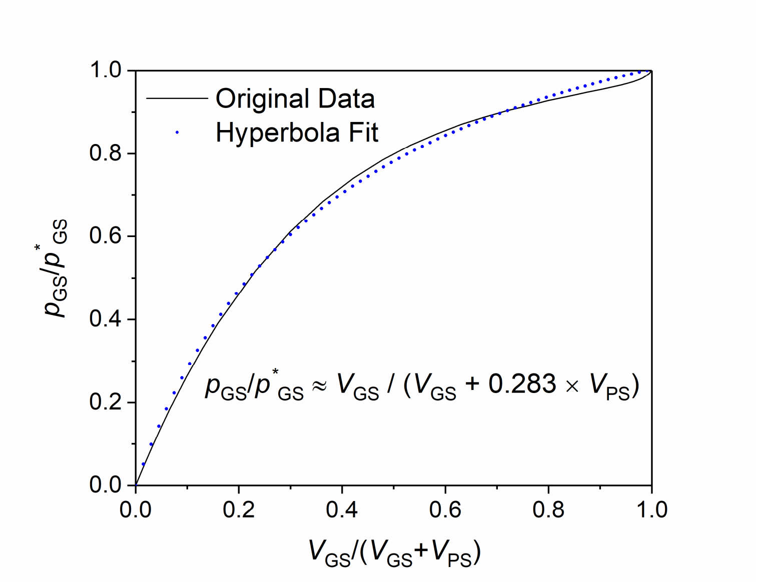

The hyperbolic curve fit, shown as the blue dotted line in the diagram above, gives the relationship between p_{GS}/p_{GS}^*, V_{GS}, and V_{PS}:

$$\frac{p_{GS}}{p_{GS}^*} \approx \frac{V_{GS}}{V_{GS}+0.283\times V_{PS}} \quad (5)$$

The volume of 2-propanol V_{PS} is assumed to be constant during the evaporation of hexane ($V_{PS}(t) = V_{PS}(0)$) because the evaporation rate of 2-propanol is about 200 times lower than that of hexane. By combining Supplementary Equation 4 and 5, the evaporation rate of the hexane is:

$$\frac{\mathrm{d}V_{GS}}{\mathrm{d}t} = \upsilon_{GS}^* \frac{V_{GS}(t)}{V_{GS}(t)+0.283\times V_{PS}(0)} \quad (6)$$

Solving this differential equation yields the analytical expression for $V_{GS}(t)$ at different V_{GS}/V_{PS} and/or N_{PE} values (Supplementary Table 1).

For N_{PE} =1 and V_{GS}/V_{PS} = 5, we obtained υ_{GS}^* = −0.0793 mL h^{-1} (Supplementary Table 1), which can be inserted in Supplementary Equation 6:

$$\frac{\mathrm{d}V_{GS}}{\mathrm{d}t} = -0.0793 \frac{V_{GS}(t)}{V_{GS}(t)+0.283\times V_{PS}(0)} \quad (7)$$

If we assume the volume of 2-propanol to be constant as mentioned above during the evaporation of hexane, we can insert $V_{PS}(0)$ = 2.5 mL in Supplementary Equation 7 to solve the differential equation and obtain an expression for $V_{GS}(t)$:

$$V_{GS}(t) = 0.708 \times \mathrm{lambertW}(8.21\times 10^8 \times e^{-0.112t}) \quad (8)$$

To demonstrate the validity of our assumption, that the volume of 2-propanol can be assumed to be constant, we calculated $V_{GS}(t)$ using $V_{PS}(t) = 2.42$ mL, which is the minimum amount of remaining volume of 2-propanol after hexane entirely evaporated:

$$V_{GS}(t) = 0.685 \times \text{lambertW}(1.54 \times 10^9 \times e^{-0.116t}) \qquad (9)$$

By plotting the solutions of $V_{GS}(t)$ of Supplementary Equation 8 and 9 together we obtain the following diagram:

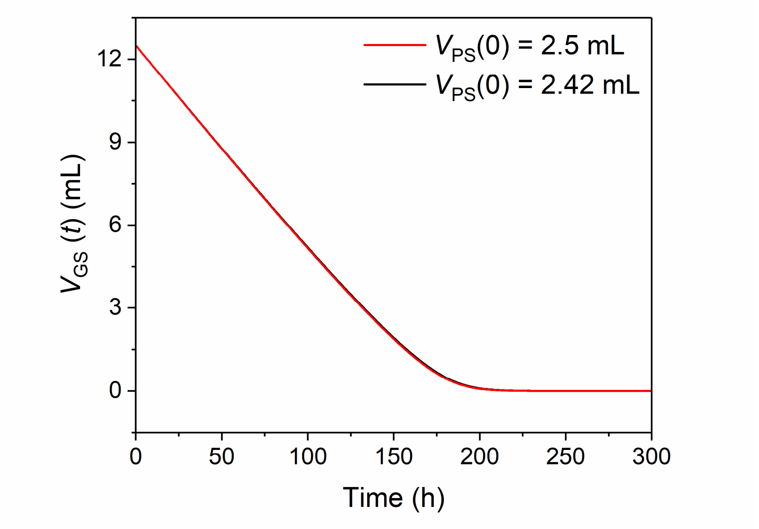

The two solutions are almost identical when using the different V_{PS} values, which supports the assumption $V_{PS}(t) = V_{PS}(0)$.

Supplementary Note 3: Correlation of $H(0)$, $H(t_1)$ and $H(t_2)$ to Δt, c_C, and P_C

We can clearly observe the starting and end point of the major growth process from the appearance of a black mesocrystal ring on the vessel wall, as shown in the left image in Figure 2a. The initial liquid level was $H(0)$, where $t = 0$. The onset of the assembly process can be estimated from $H(t_1)$, and the assembly process terminates at $H(t_2)$. With a cylindrical beaker, the total volume $V(t)$ in the vessel is proportional to the height of the liquid level $H(t)$. The total volume is estimated from the sum of the volume of hexane and 2-propanol as $V(t) = V_{GS}(t) + V_{PS}(t)$. The excess volume of the hexane–2-propanol mixture is insignificant (less than 4‰ at 298 K).[6] The time-dependent height of the liquid and the total volume in the cylindrical vessel during evaporation can be expressed as:

$$\frac{H(0)}{H(t)} = \frac{V(0)}{V(t)} = \frac{V_{GS}(0)+V_{GS}(0)/(V_{GS}/V_{PS})}{V_{GS}(t)+V_{GS}(0)(V_{GS}/V_{PS})} \qquad (10)$$

and $V_{GS}(t)$ can be expressed in terms of the known quantities $V_{GS}(0)$, $H(0)$, $H(t)$, and V_{GS}/V_{PS}:

$$V_{GS}(t) = V_{GS}(0)\frac{H(t)(1+V_{GS}/V_{PS})-H(0)}{H(0)(V_{GS}/V_{PS})} \quad (11)$$

Herein, $V_{GS}(t_1)$ and $V_{GS}(t_2)$ can be obtained from $H(0)$, $H(t_1)$ and $H(t_2)$ using Supplementary Equation 11. The time of the assembly process ($\Delta t = t_2 - t_1$) can be obtained from the solutions of Supplementary Equation 6 in Supplementary Table 1.

The critical concentration $c_C = c(t_1)$ at the onset of the major mesocrystal growth stage is obtained from:

$$c_C = c(t_1) = c(0)\frac{V(0)}{V(t_1)} = c(0)\frac{H(0)}{H(t_1)} \quad (12)$$

The free OA volume fractions x_{OA} at the onset of mesocrystal growth are always less than 2.1% (Supplementary Table 2), which suggests that the free OA has a negligible influence on the polarity of the dispersion media.

Supplementary Note 4: Up and down scaling and minimization of assembly time

The evaporation-driven poor-solvent enrichment method has been be scaled down or up and generated mesocrystals from dispersions with initial volumes ranging from 1.5 mL (sample #**10** with 4.5 mg NC) to 120 mL (sample #**11** with 360 mg NC). It should be noted that it is important to control and optimize the ratio of vessel opening area to initial volume $A_O/V(0)$, and the ratio of the total amount of NC to the bottom area of the vessel m_{NC}/A_B. For preparing mesocrystals with regular morphologies, our studies suggests that the ratio m_{NC}/A_B < 10 mg cm^{-2}.

Reducing the V_{GS}/V_{PS} ratio to 2.4, covering the vessel with 4 layer of PE, and increasing $c(0)$ to 5.3 mg mL^{-1} (the blue arrow marked in Supplementary Figure 12), results in a system where mesocrystal assembly will initiate after about 50 hours of evaporation and will be complete after about 70 hours. The produced mesocrystals (sample #**12**, Supplementary Figure 13) show a similar size and morphology compared with the mesocrystals in sample #**9** (Supplementary Figure 10c), even though the experimental time was 200 h shorter. Hence, dispersions with similar Δt, c_C, and P_C can generate mesocrystals with similar sizes and quality by the EDPSE method although the time to onset of assembly differ significantly.

Supplementary Note 5: Peak assignment of SAXS profile and structure analysis

As shown in Supplementary Figure 16a, $d_{(111)} = 2\pi/q_{(111)} = 13.2$ nm for sample #**D1**. Then the unit cell parameter a of sample #**D1** can be given as:

$$a = d_{(111)} \times \sqrt{3} = 22.9 \text{ nm}$$

Similarly, we obtain $a = 23.7$ nm for sample #**D2** ($d_{(111)} = 13.7$ nm) and 22.3 nm for sample #**D3** ($d_{(111)} = 12.9$ nm). It becomes evident that the contraction in the PS enriched systems #**D1** and #**D3** leads to lower distances between the NCs compared to the pure GS system #**D2** due to an increasing polarity and the contraction of the non-polar alkyl chains of OA. Due to the lack of OA in sample #**D3**, the final value of $d_{(111)}$ is lower than in sample #**D1**.

Since the NC core size is fixed, the different a values are caused by the various lengths of the OA double layer d_{OA} which can be varied by different solvent polarities and surface coverages. Assuming the NC is a perfect cube with an edge length of $d_{\text{NC}} = d_{\text{TEM}} = 10.8$ nm, the packing efficiency (or volume ratio) of the NC core φ_{NC} can be written as:

$$\varphi_{\text{NC}} = \frac{V_{\text{NC}} \times 4}{V_{\text{cell}}} = \frac{{d_{\text{NC}}}^3 \times 4}{a^3}$$

We get $\varphi_{\text{NC}} = 0.42$, 0.38 and 0.45 for samples #**D1**, #**D2** and #**D3**, respectively. In the real case, V_{NC} is slightly smaller than d_{NC}^3, due the truncated corner (Supplementary Figure 1).

For a perfect *fcc* lattice where the NCs are in an edge-to-edge packing (Supplementary Figure 17a), the cell parameter a can be given in terms of d_{NC} and $d_{\text{OA}} = 2$–3 nm:

$$a = (d_{\text{NC}} + d_{\text{OA}}) \times 2 = 25.6 - 27.6 \text{ nm}$$

which is larger than the experimental a value. Besides, the edge-to-edge packing is also not energetically favorable. The packing efficiency is $\varphi_{\text{NC}} = 0.24$–0.30.

A more stable configuration is that the NCs are in contact by a face-to-face manner where the NCs are tilted by 45° in x, y, and z directions (Supplementary Figure 17b).[7] For such configuration, the NCs are in a partly overlapping face-to-face arrangement along the [111] superlattice direction, as the blue and red nanocubes indicate in Supplementary Figure 17b. The value of $d_{(111)}$ and a are:

$$d_{(111)} = d_{\text{NC}} + d_{\text{OA}} = 12.8 - 13.8 \text{ nm}$$

$$a = d_{(111)} \times \sqrt{3} = 22.2 - 23.9 \text{ nm}$$

Those values agree very well with the experimental data from Supplementary Figure 16. The packing efficiency is 0.37–0.46 for such configuration, which is consistent with the φ_{NC} values we obtained above.

Moreover, the most efficient packing for NCs is simple cubic (*sc*), the packing efficiency of sc lattice for d_{NC} = 10.8 nm and d_{OA} = 2–3 nm is 0.48–0.60.

Supplementary References

1. Zhang L., He R., Gu H.-C. Oleic acid coating on the monodisperse magnetite nanoparticles. *Applied Surface Science* **253**, 2611-2617 (2006).

2. Kubík L. u., Zeman S. Permeability of Polymeric Packaging Materials. *Technical Sciences* **10**, 33-34 (2007).

3. Dortmund Data Bank. Oldenburg, Germany: DDBST-Dortmund Data Bank Softer & Seperation Technologh GmbH; 2015.

4. Perry's R. H., Chilton C., Kirkpatrick S. Chemical engineers handbook. *Chemical engineers handbook* (1999).

5. Abrams D. S., Prausnitz J. M. Statistical thermodynamics of liquid mixtures: A new expression for the excess Gibbs energy of partly or completely miscible systems. *AIChE J.* **21**, 116-128 (1975).

6. Morrone S. R., Francesconi A. Z. Excess volumes of (hexane or cyclohexane + propan-2-ol or butan-2-ol) at the temperatures (288.15 and 298.15) K. *J. Chem. Thermodyn.* **28**, 935-940 (1996).

7. Quan Z., Loc W. S., Lin C., Luo Z., Yang K., Wang Y., *et al.* Tilted face-centered-cubic supercrystals of PbS nanocubes. *Nano Lett.* **12**, 4409-4413 (2012).

8. Young K. L., Personick M. L., Engel M., Damasceno P. F., Barnaby S. N., Bleher R., *et al.* A directional entropic force approach to assemble anisotropic nanoparticles into superlattices. *Angew. Chem. Int. Ed.* **52**, 13980-13984 (2013).
