# Supplementary material for: Tunable assembly of truncated nanocubes by evaporation-driven poor-solvent enrichment
Source: Nat Commun. 2019 Sep 17;10:4228. doi: 10.1038/s41467-019-12237-y (PMC6748999; doi:10.1038/s41467-019-12237-y)
Supplement: Supplementary file 3 — Description of Additional Supplementary Files [file 41467_2019_12237_MOESM3_ESM.pdf]

Description of Additional Supplementary Files

File Name: Supplementary Movie 1

Description: This movie was composed by 24 images of the single mesocrystal in Fig. 1d rotating on the X-ray diffractometer at different rotating angle Phi (15° per step). This single crystal was collected from #9. The top-right inset showed the corresponding SC-XRD pattern.
